# Systemic Case Formulation, Individualized Process Monitoring, and State Dynamics in a Case of Dissociative Identity Disorder

**DOI:** 10.3389/fpsyg.2016.01545

**Published:** 2016-10-20

**Authors:** Günter K. Schiepek, Barbara Stöger-Schmidinger, Wolfgang Aichhorn, Helmut Schöller, Benjamin Aas

**Affiliations:** ^1^Institute of Synergetics and Psychotherapy Research, Paracelsus Medical UniversitySalzburg, Austria; ^2^Department of Psychology and Pedagogics, Ludwig Maximilians UniversityMunich, Germany; ^3^Department of Psychosomatics and Inpatient Psychotherapy, Paracelsus Medical University, Christian Doppler University HospitalSalzburg, Austria

**Keywords:** idiographic system modeling, systemic case formulation, real-time monitoring, therapy feedback, Synergetic Navigation System (SNS), personality states, borderline personality disorder, dissociative identity disorder

## Abstract

**Objective:** The aim of this case report is to demonstrate the feasibility of a systemic procedure (synergetic process management) including modeling of the idiographic psychological system and continuous high-frequency monitoring of change dynamics in a case of dissociative identity disorder. The psychotherapy was realized in a day treatment center with a female client diagnosed with borderline personality disorder (BPD) and dissociative identity disorder.

**Methods:** A three hour long co-creative session at the beginning of the treatment period allowed for modeling the systemic network of the client's dynamics of cognitions, emotions, and behavior. The components (variables) of this idiographic system model (ISM) were used to create items for an individualized process questionnaire for the client. The questionnaire was administered daily through an internet-based monitoring tool (Synergetic Navigation System, SNS), to capture the client's individual change process continuously throughout the therapy and after-care period. The resulting time series were reflected by therapist and client in therapeutic feedback sessions.

**Results:** For the client it was important to see how the personality states dominating her daily life were represented by her idiographic system model and how the transitions between each state could be explained and understood by the activating and inhibiting relations between the cognitive-emotional components of that system. Continuous monitoring of her cognitions, emotions, and behavior via SNS allowed for identification of important triggers, dynamic patterns, and psychological mechanisms behind seemingly erratic state fluctuations. These insights enabled a change in management of the dynamics and an intensified trauma-focused therapy.

**Conclusion:** By making use of the systemic case formulation technique and subsequent daily online monitoring, client and therapist continuously refer to detailed visualizations of the mental and behavioral network and its dynamics (e.g., order transitions). Effects on self-related information processing, on identity development, and toward a more pronounced autonomy in life (instead of feeling helpless against the chaoticity of state dynamics) were evident in the presented case and documented by the monitoring system.

## Introduction

Psychopathologies are marked by cognitive, emotional, and behavioral deviations that form a major burden on the life of clients and simultaneously hinder the cure of these pathologies. Eventually, the tools needed to bring along psychotherapeutic progress are affected by syndromes, making that what is to be cured an integral part of the cure itself. This circularity makes it understandable that patients with severe pathologies, as, e.g., personality disorders, dissociative identity disorder, or schizophrenia, generally remain in treatment for longer and have a poorer projected outcome. It is especially in such severe cases, where therapists face clients with troubled focus when in dialog, impaired memory, or labile emotional conditions which affect therapeutic sessions and render some psychological techniques unfeasible. However, when therapy (i) is marked by a focus on resources rather than pathologies, (ii) makes use of highly individualized techniques, (iii) entails systemic and systematic mapping of the psychological landscape of a client, and (iv) allows for high-resolution monitoring of the ongoing processes therein, one can hypothesize that a client will feel apprehended, better understand seemingly volatile processes and enter a sense of agency. Thereby, one actually turns around the above described problematic circularity and utilizes it for a better psychotherapeutic progress, also in severe cases.

In the present paper we report on a client with dissociative identity disorder and co-morbid borderline personality disorder that followed a therapeutic approach that has earlier been described as synergetic process management (Haken and Schiepek, 2010; Schiepek et al., 2015, 2016). Making use of an online monitoring system (Synergetic Navigation System, SNS) which allows for therapeutic feedback and management, synergetic process management entails

- a resource-focused interview,
- the development of an idiographic system model, covering psycho- and socio-dynamics, from which one derives
- an individual process questionnaire for daily online monitoring,
- regular therapeutic sessions with feedback on basis of the current data-profile (continuous cooperative process control), and
- out-patient aftercare with the Synergetic Navigation System as bridging technology.

The aim of this case study is to present in detail the applied therapeutic procedure and confirm the hypothesis that an individualized, systemic, feedback-driven, monitoring-based therapy approach is not only a viable method for severe psychopathologies, but also an “interscholastic” approach, independent of psychotherapy “schools” and confessions.

## Methods

### The client

The client (Mrs. A.) was a middle-aged German woman with long-term experience as employee of a tax accountant office. After a number of subsequent heterosexual private relationships, in which she was exposed to continuous violence and at times sadistic behavior by her partners, she lived in a lesbian partnership with a same aged woman. When entering the day-treatment program, she had been on sick leave for a longer period and had previously completed an in-patient treatment at the same clinic 1 year before. Despite many traumatizing events since childhood (e.g., mobbing in school, social isolation in puberty, violence in relationships), she managed to uphold 15 years of dedicated office work for the same employer and lead a rather orderly life. The takeover of “her” accountant office by an external firm and the accompanying loss of job, marked the beginning of a crisis for Mrs. A. New foci in her life became automutilation, changing dissociative personality states with mutual amnestic nature, subsequent chronologic disorientation and insecurity about her behavior in these separate states, lack of concentration, and feelings of de-realization (e.g., certain situations with diffused incidence of light possibly carried Mrs. A. back to childhood). At times, amnesias could block memory of entire days. The diagnostic exploration of these experiences through the SKID-D (Gest et al., 2000) suggested a “complex dissociative disorder” (dissociative disorder not otherwise specified, F44.9), while fulfilling the criteria of the DSM-5 diagnoses of a borderline personality disorder (301.83) and other specified dissociative disorders (300.15). According to the nomenclature of Nijenhuis (2016; Nijenhuis and van der Hart, 2011), Mrs. A. could be diagnosed with a “minor dissociative identity disorder,” in terms of a tertiary structural dissociation. Besides these formal clinical attributes, she was concerned whether she would be able to have a healthy life again and form an own, congruent identity. She described herself as a “blank sheet of paper,” ready for anyone to come along and write on her as he or she pleases—a metaphor for the assaults and abuses she had experienced.

### Resource-focused interview

In a resource-focused interview, therapist and client first assessed the major challenges of the client's life, taking into account that topics need to be changeable and goals reachable. Mrs. A. reported that she would like to “be able to distance herself from loud noises and intrusive voices in her direct surrounding” (note that these are real life situations as e.g., in the office and not psychotic hallucinations). Furthermore, she wanted to have “stability in life” and “find a job,” the latter she would dismiss throughout therapy as too big a burden. Secondly, therapist and client explored in a dialogical fashion the resources of the client, which were in a third step rated for their current, desired and potential manifestation. As resources, Mrs. A. experienced the felt love for her partner, she enjoyed listening to music (as means to distance herself from unpleasant sounds around her), knitting, liked herself for having a dry and at times satirical humor, being a good listener, a general patience and endurance. Also, she saw herself competent in terms of being a reliable person, having a pool of positive memories (e.g., odors from her childhood) and her “head-cinema”—a technique she invented for herself in which she uses aggressive fantasies featuring the perpetrator of intrusive sounds, in order to channel off her disturbance. Even though some of these resources might appear questionable, the condition of Mrs. A. at the beginning of therapy was such that she truly felt these were capacities and resources that help her in life.

The rationale behind applying a resource-focused interview at the beginning of treatment is manifold. Foremost, it sets a positive antipode to classical psycho-diagnostics, which almost exclusively focus on pathology. Many clients—and so did Mrs. A.—experience the positive tone and direction of such an interview as a relief and a reminder of one's strengths, creating a positive mindset. A second benefit of the rather open and loosely structured resource interview, is its function as a prelude to the idiographic system model, priming the search for psycho-social relevant variables in a client's life.

### Idiographic system model

As the chronological next part of the synergetic process management, one tries to produce a list of important psychological and social variables of the client in a second interview. Starting off with e.g., a general picture of the client's life in the last couple of months, the therapist takes note throughout the interview of important factors such as psychological problems, problem-solving methods, coping strategies, and impact on social life. These notes will form the basic components of the to be developed idiographic system model (Schiepek, 2003; Schiepek et al., 2015). Therefore, virtually any topic of importance to the client can be part of the interview and enter the system. It is advisable to try to capture the actual terms of the client's language, in order for client and therapist to achieve mutual understanding and produce the client's very own individual model. After the interview, all variables are checked for their terminology and content, to make sure that the client can find him or herself therein. It is important that the components are expressed as variables that can change throughout time. In a perfect case, therapist and client manage to capture all important bio-psycho-social aspects of the client's life, incorporating cognitions, emotions, motives, behavior, physiological states and more, yet by using the client's own language and terminology.

**Figure 1:**
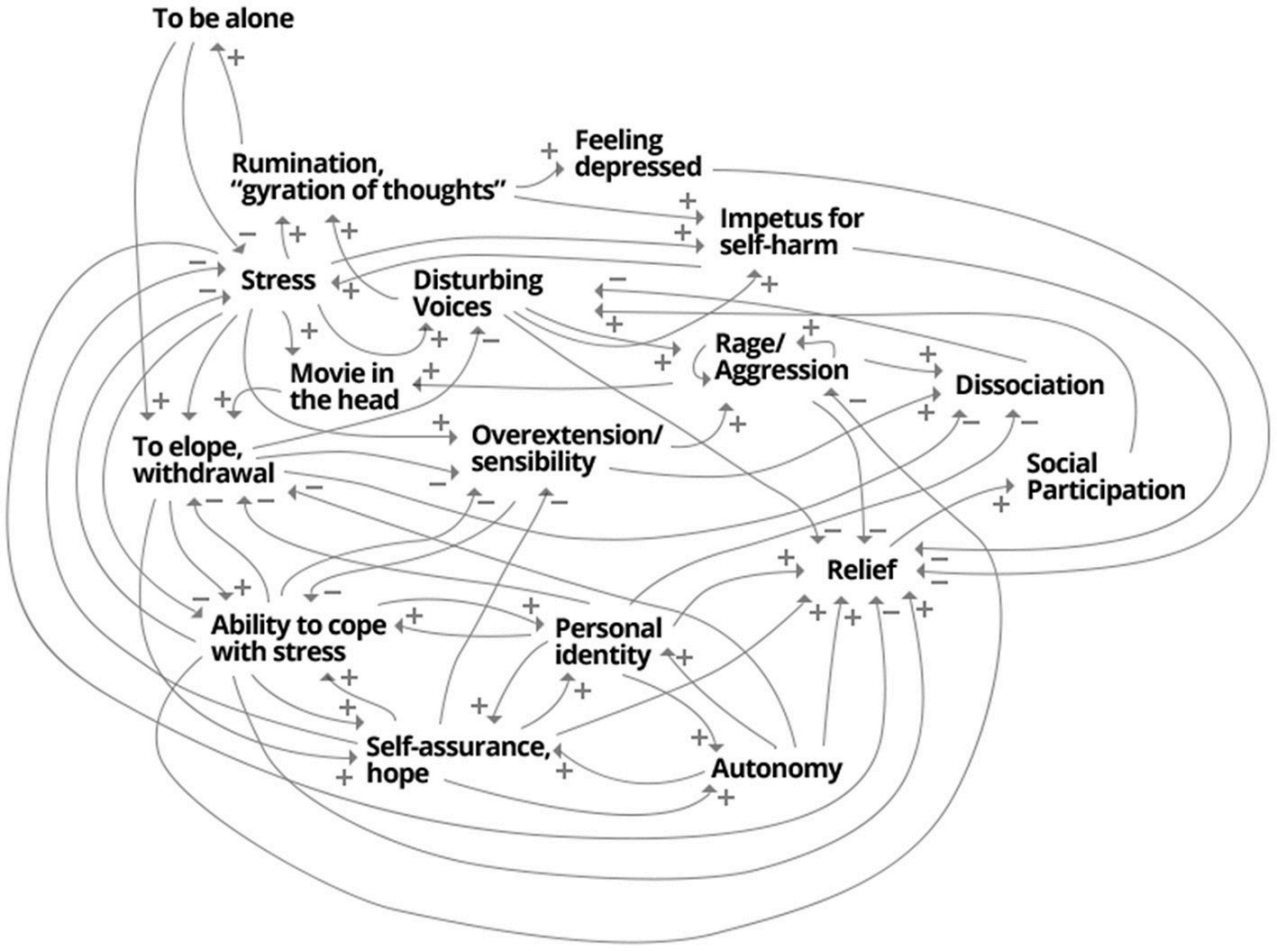
**The idiographic system model of Mrs. A**. A synopsis of psycho- and socio-dynamical aspects of the patient's experiences.

Subsequently, the inter-connections of these variables are mapped, creating a personal landscape of relevant aspects of the client's mental functioning—the idiographic system model (ISM). Using a flipchart, a variable A is written down and the list is checked for other variables that are connected to it. Writing down a second connected variable B, both are linked with an arrow and a + or − symbol, indicating whether there is a positive relation (same-directed: increase in A leads to increase in B and decrease in A leads to decrease in B) or a negative relation (opposite-directed: increase in A leads to decrease in B and decrease in A leads to increase in B). As can be seen in Figure 1, Mrs. A. e.g., described that an increase in “dissociation” is accompanied with a decrease of the distraction through “disturbing voices,” indicated by a — between the two variables. In contrast and symbolized by a +, the more “rage/aggression” she experiences, the more she needs to switch on her “movie in her head (head-cinema),” and a decrease in aggression makes that coping-mechanism less necessary.

Mrs. A. managed to create her complete ISM in a session of 3 hours, being in a, as she described it, “focused flow” only interrupted by a short break. In contrast to her everyday life experiences of dissociative absences, she managed to keep up her concentration fairly uninterrupted through this intensive session. She reported to find herself very well represented by the model and brought a small copy of the model to almost all therapeutic sessions, referring to it as “the map of my soul”. Often, the model helped her to better understand patterns of her own behavior, and how in a systemic fashion, cognitions, emotions and behaviors could trigger each other. In a second step, the client attributed many of the variables to be differentially prominent in separate personality states. That understanding was the basis for seeing patterns, that before had presented themselves as volatile and erratic alternations of these states. In contrast to her everyday life experience, the ISM functioned as a systemic and systematic synopsis of her psychological and social life, making amnestic separations of important aspects visible. Consequently, this understanding allowed trauma-focused therapy and intensive work directed toward the different states (Nijenhuis, 2015, 2016).

### Individual process questionnaire and monitoring

After completion of the idiographic system modeling, therapist and client made use of the editor in the Synergetic Navigation System (SNS) and created an individual questionnaire (this happened two sessions after the ISM, because the intermediate session had been marked by lack of concentration of Mrs. A.). Table 1 shows the items of the personalized questionnaire, based on the variables of the ISM. The client filled in each item on a visual analog slider, ranging from “not at all” to “very much,” which is subsequently scored on a scale from 0 to 100.

**Table 1:** **The 18 items of the individual questionnaire of Mrs. A**.

I Stress and Coping (state-cluster “child”, EP, corresponds to factor I of the individual questionnaire)
1. Today, I experienced stress …
2. Today, I had to activate my “head-cinema“ (“movie in the head”) …
3. Today, I zoomed out - dissociated …
4. Today, it was important to me to be alone …
5. Today, the depression carried me away …
6. The impulse to hurt myself was today …
7. Today, I ruminated …
8. The intrusive voices were today …
9. My level of aggression was today …
10. My level of anger was today …
11. Today, I felt overwhelmed …
12. My need for distancing myself from others was today …
II Positive goals and development of identity (state-cluster “adult”, ANP, corresponds to factor II of the individual questionnaire)
13. Today, I felt resilient and able to cope with stress …
14. My feelings of inner security were today …
15. My feelings of independence were today …
16. The sense of my own inner identity was today …
17. Today I had a sense of relief …
18. Today, I took part in social life …

Match the 18 variables of her ISM, as shown in Figure 1, separated into two factors. The client answered these items daily via the online monitoring system SNS. Each question is scored by a visual analog slider (VAS), ranging from 0 to 100 and extrema of “not at all” to “very much” (where applicable).

**Figure 2:**
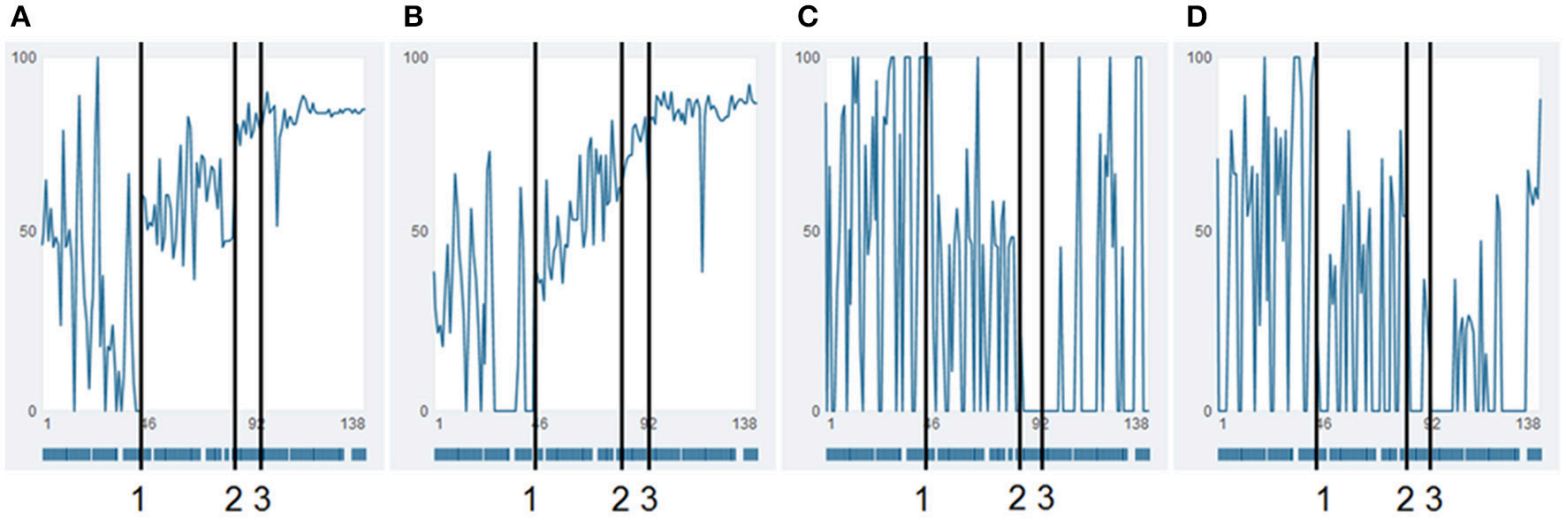
**Raw data time series of four items**. Time series of the raw data of items “resilience/ability to cope with stress” **(A)**, “autonomy” **(B)** (both factor II), “withdrawal” **(C)**, and “stress” **(D)** (both factor I). Numbers 1 and 2 indicate time points, where not only the absolute values of the time series suddenly shift, but also new dynamic patterns emerge. Such transitions and re-stabilizations are generally denoted in complex systems theory as order transitions. It is noteworthy, that even though the shifts present themselves as sudden tipping points, these are not necessarily caused by a singular event, but can be the result of a continuous underlying process, affecting the complete system of the patient, such as e.g. ongoing psychotherapy. Number 3 is the day of dismissal from the day-treatment clinic. **(A,B)** show extreme, irregular fluctuations at beginning until flag 1. Phase two of these items is marked by similar fluctuations, however with less extreme values. The items in Figures **(A,B)** show a general absolute increase with simultaneously decreasing fluctuations (dynamic complexity), and with specific pattern transitions. The item “resilience” shows the pattern of self-similarity; the whole process has a similar “Gestalt” as a shorter part of the time series, between flag 1 and 3.

**Figure 3:**
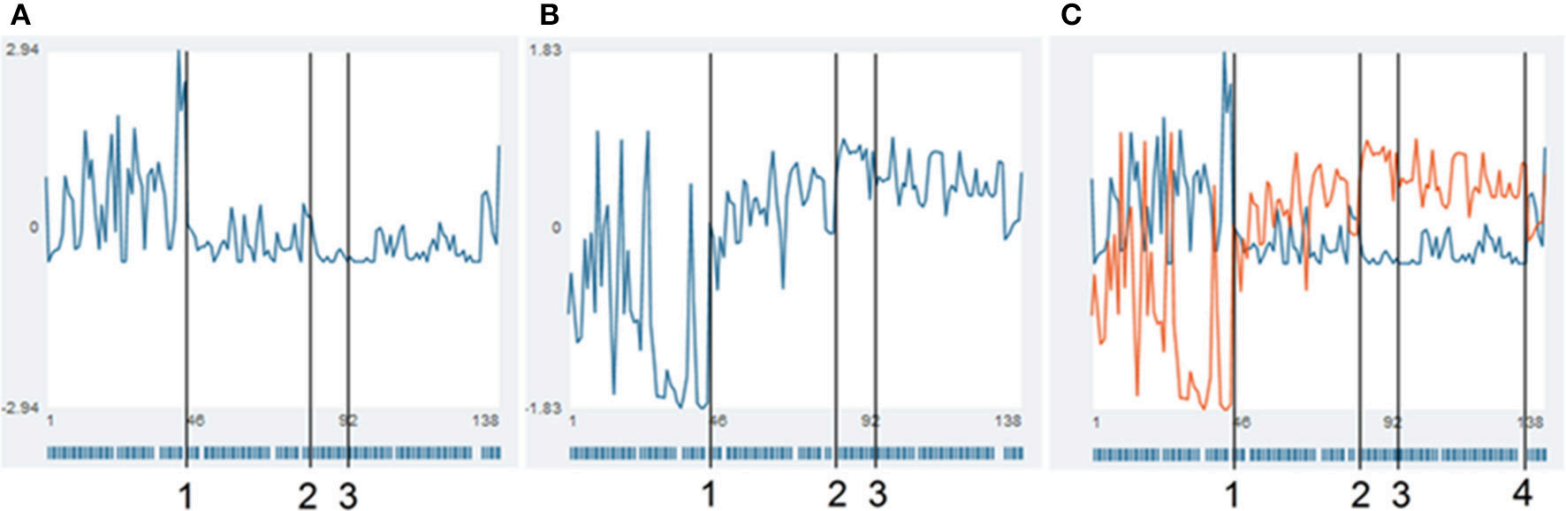
**Time series of two factors, combining all items of Mrs. A**. Time series of the superordinate factors of Mrs. A. Each time-point represents the standardized mean of the respective items per factor. **(A)** Stress and coping (“child”-states, factor I), **(B)** positive goals and development of identity (“adult”-states, factor II), **(C)** superposition of the time series (blue, factor I and orange, factor II). The bar-code like stripes at the bottom of each diagram depict the days Mrs. A. used the commentary function of the SNS (the diary entries are shown in the SNS when the mouse is moved over a time point). Clearly visible are the alternating extrema of the two state-clusters in the first half of the therapy (until flag 1). Vertical lines (1) and (2) indicate order transitions in the patterns of the time series, flag 3 defines the day of dismissal from the day-treatment clinic, at (4), a personal crisis in the client's private life occurred.

The actual formulation of the items is thereby dictated by the client, the therapist merely advises and live-edits the questionnaire online. An example of the time series of four items is given in Figure 2. Mrs. A. classified the items into two factors, which interestingly turned out to be in accordance with two dominant states (comprised of sets of sub-states); as can be seen in Figure 3A, a “child-state” (in terms of the conceptualization of a structural dissociation: emotional personality aspects, EP's) and an “adult state” (“apparently normal personality aspects,” ANP's; Figure 3B). During the first third of the monitoring process, these two states showed a rather alternating dynamic, excluding the presence of each other (Figure 3C, until flag 1). Mrs. A. filled in this personalized questionnaire online daily, not missing a single day.

### Visualization and analysis methods

#### Raw-data resonance diagrams

**Figure 4:**
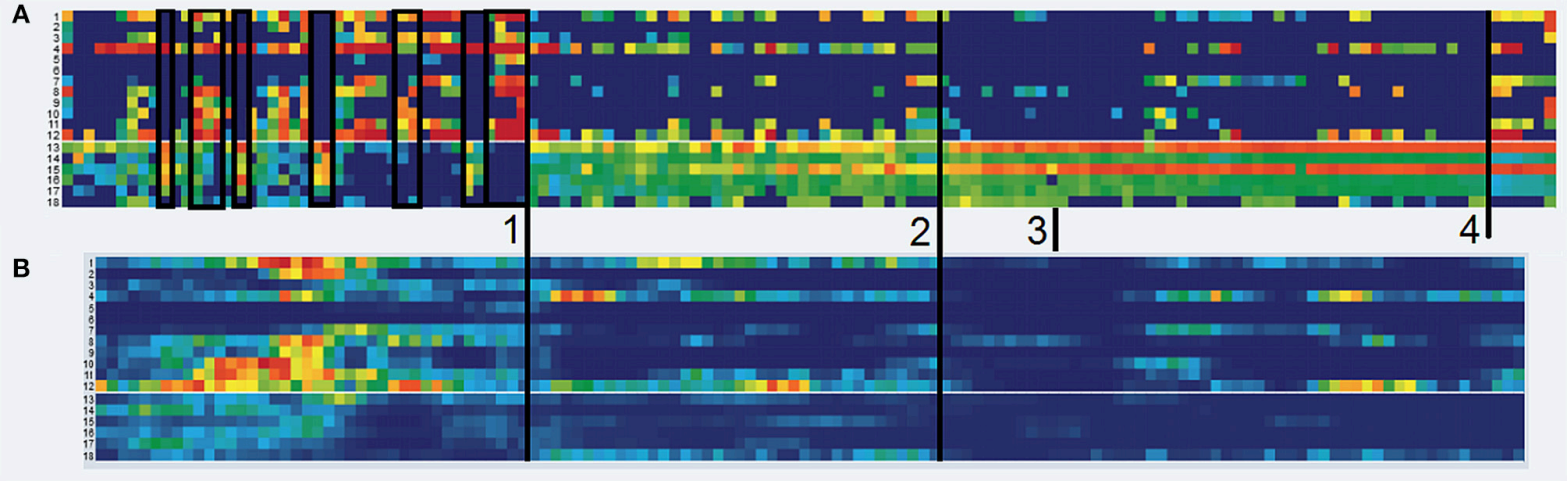
**Raw data resonance diagram and complexity resonance diagram**. **(A)** Raw-data Resonance Diagram. Depicted are the manifestations of the time series per item (row). The original scores ranging from 0 to 100 are transformed to a continuous rainbow-like color-scale ranging from blue (0) via turquois, green and yellow (increasing medium scores) to red (100). The slim white demarcation between row 12 and 13 discriminates the factors (see Table 1). Black frames underline periods of alternating item scores and manifestation of Mrs A.'s states in the first third of the monitoring period (until flag 1). **(B)** Complexity Resonance Diagram. Depicted is the dynamic complexity in overlapping time windows (window width = 7 days). The maximum score of the dynamic complexity is depicted by a full red pixel, while all other values are graded according to that maximum (red = high, yellow = medium, blue = low complexity). The cluster of high dynamic complexity occurs especially in the items of factor 1 before flag 1, corresponding to the intensely fluctuating and mutually exclusive states.

In order to grasp the process and therapeutic changes, the time series of each item can be plotted in one diagram each (comp. Figure 2). In these diagrams, each item or factor has to be drawn as a single line, making it difficult to get a synoptic overview of the 18 items Mrs. A. filled in daily. In order to get such synopsis, one can use the visualization of raw-data resonance diagrams in the SNS, which allows for visualization of all items in a single diagram. Here, the manifestation of each item is expressed by a color-coded scale. As can be seen in Figure 4A, all items are depicted as separate rows, while each column represents a single day. Each cell represents the response of the client on that respective day on a scale ranging from low (blue) via medium (green and yellow) to high scores (red).

#### Complexity resonance diagrams

In order to analyze the changing complexity of each item, a measure called “dynamic complexity” can be calculated in a moving window of freely selectable width (here: 7 days) and displayed as variation in time for each item of a questionnaire. Dynamic complexity is composed of a fluctuation and a distribution value (Schiepek and Strunk, 2010). Fluctuation is computed for each window, using the number of directional changes of the values and the size of each daily in-/decrease. Distribution is a parameter that increases when the values of a time series within a respective window make use of the complete spectrum of the scale and if the scores are evenly distributed across the scale. The resulting dynamic complexity can be thought of as an own time series per item. In the complexity resonance diagram (CRD), this time series of the dynamic complexity is expressed as rainbow colored pixels, while each pixel corresponds to the amount of complexity within a specific item and within a certain time window (see Figure 4B). Ranging from no dynamic complexity (blue) to strong complexity (the maximum of each matrix is depicted as deep red), these CRDs thus allow for identification of periods with strong simultaneous change and also periods of stable scores across the complete underlying questionnaire.

#### Moving correlation matrices

**Figure 5:**
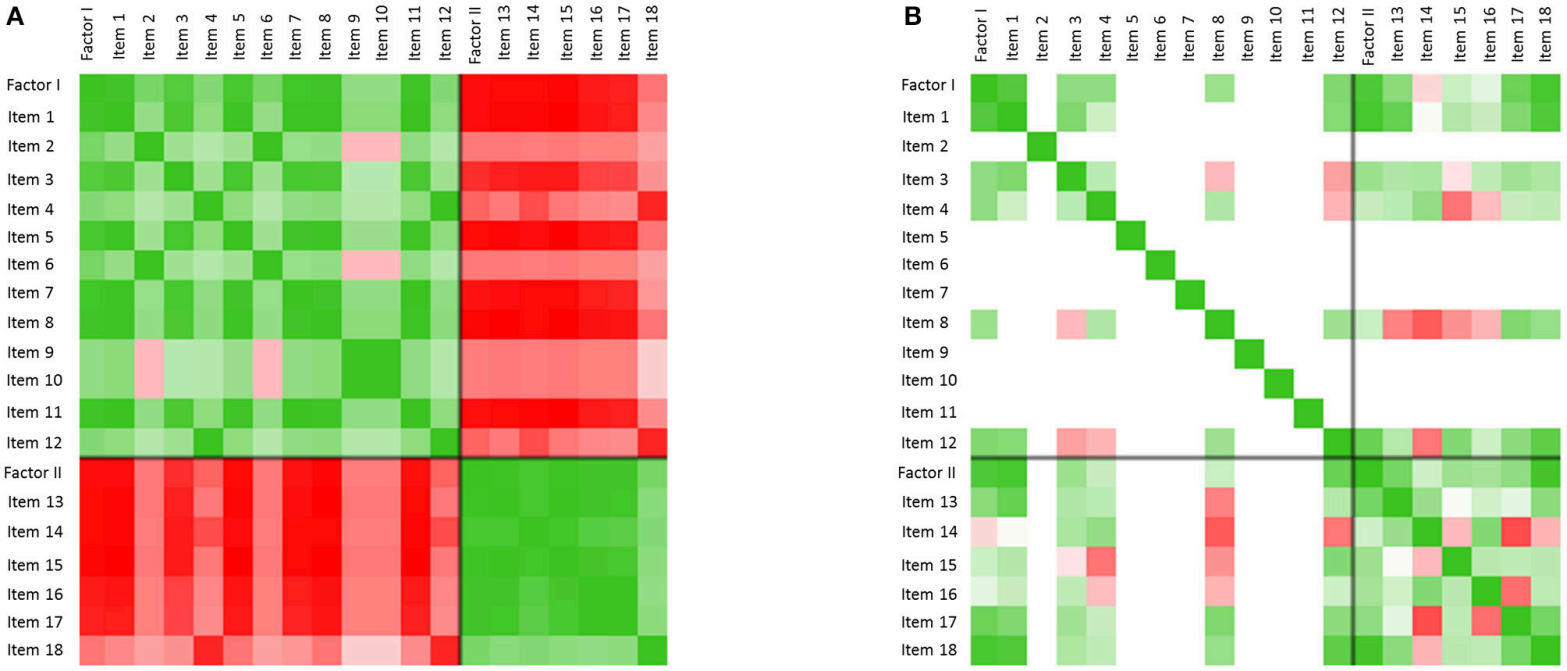
**Inter-item correlation matrices. (A)** Color-coded inter-item correlation pattern characterizing the first third of the monitoring period (before flag 1). Each cell depicts the correlation of a respective item with another item on a gradual green (positive correlation values, 0 < *r* < 1) or red (negative correlation values, −1 < *r* < 0) scale (white cells correspond to a correlation of 0). The black cross in each matrix differentiates items from factor I and factor II. The left matrix (*t* = 41–47) is characterized by high positive within-factor item correlations and negative between-factor item correlations (green and red blocks per factor). **(B)** Only some days later (*t* = 49–56), but after the first order transition of the therapy (occurring at flag 1), this pattern dissolved. The change of correlation patterns concurs with Mrs. A.'s reports of increasing integration of her separate personality states throughout the therapeutic process.

The items of a process questionnaire (here: two factors and their respective items) can be correlated in moving time-windows. In it, all inter-item Pearson's *r* correlations are calculated in shifting correlation matrices for a time window of free choice (here: 7 days). Each cell depicts the correlation of a respective item with another item on a graded green (positive) and red (negative) scale, with intensities of green corresponding to positive correlations, white corresponding to 0, and intensities of red corresponding to negative correlations. Figure 5 depicts to this method.

#### Recurrence plots

Psychological patterns such as alternating states reoccur over and over in Mrs. A.'s life. One way to visualize the similarity of dynamics in a time series is the method of recurrence plots (Eckmann et al., 1987; Webber and Zbilut, 1994; Haken and Schiepek, 2010, S. 395–401). In these, the values of a time series are transposed to vectors in a phase space defined by time-delay coordinates. Since sequences of measurement points are transformed into time-delay coordinates, it is possible to depict the reoccurrence of the measurement sequences (vector points) and thereby to identify the similarity or dissimilarity of patterns throughout time. The Euclidic distance between vector points is directly transferred to a color scale (blueish colors are “recurrent,” and warm colors from yellow to red are “transient”). In **Figure 7**, the dynamics of the items “resilience/ability to cope with stress” (**A**), “autonomy” (**B**) (both factor II), “withdrawal” (**C**), and “stress” (**D**) (both factor I) are analyzed using the method of colored recurrence plots. The advantage of this technique becomes especially evident when looking at the raw value of these four items, as shown in Figure 2. The three periods suggested through the recurrence plot would not be as obvious in the time series itself. Identifying such periods might however be important information for therapeutic understanding and intervention, only accessible through a holistic conceptual approach which combines high-resolution monitoring techniques and appropriate analysis techniques.

## Results

### State dynamics

During the first third of the complete monitoring period and almost the first half of the therapy, the two states described above (a “child-state,” corresponding to factor I, and an “adult state,” corresponding to factor II, see Table 1) showed rather alternating dynamics, excluding the presence of each other (Figure 3C, until flag 1). This pattern of alternating states is visible through the volatile contrasts between items 1 and 12 of factor I and items 13 to 18 of factor II, for the period until mark 1. In the raw data resonance diagrams these contrasts occur in blocks, as is underlined by black frames in Figure 4A. In this period, the dynamic complexity of most of the factor I items realizes the most pronounced values (Figure 4B), corresponding to the volatility and extremely erratic fluctuations of the components of her cognitive-emotional system (comp. Figure 2).

Figure 5A shows this pattern of alternating and mutually exclusive states in terms of inter-item correlations. The correlation matrix refers to inter-item correlations calculated for the therapy days 41–47 (window width = 7 days). The two prominent green blocks of the matrix (upper left green block: factor I, lower right green block: factor II) underline the high correlation of the items within the factors, which is realized during almost the complete first half of the therapy in a similar way. During this period, the correlation of the items with respect to the items of the other factor is highly and consistently negative, as the red blocks in Figure 5A show. This asynchrony of the two factors mirrors the exclusive alternation of the respective personality states during the early state of the therapy.

### Order transitions

Before and throughout the first half of the therapy, Mrs. A's case was marked by a pattern of (roughly) daily interchanging personality states. At a certain point during the therapy (marked as time point number 1 in all applicable figures) that alternating pattern disappeared. That is when the client abolished her previous goal to soon enter the first labor market again (see resource-focused interview), which she described as a great relief. An attractive job offer by a friend had triggered days of ambivalent feelings, ambiguity, and inner conflicts (see theory-compliant fluctuations just before flag 1 in Figure 3). Instead of her earlier behavior of allowing others to—as she herself put it—“whip her into” new situations, she was capable of allowing herself to turn down the offer. She experienced this decision as big liberation, listening to her inner voice. A process enabled by previous work on traumata and states, in which the creation of the idiographic system model and thereby a better understanding of mechanics of her state dynamics played a major role (e.g., understanding the relation between voices experienced as disturbing and incidences of traumatizing violence in earlier relationships). Mrs. A.'s entry in her SNS-based electronic diary at this order transition said: “…I have the feeling of being myself again (…) the last couple of days were unpleasant and painful. (…) Decisions for the time after the hospital stay have been made, which are better for me. I want to make peace with myself; that does not always work out, but is so important!! Because the last years I always tried and worked on myself to find work again, but always felt so much stress and pressure (…) and that is not how things work!! My switches are set differently (…), in order to have some room for peace and let the stress go and to think about, what I really want to do and what I could work. (…) I will not surrender, to nothing and nobody!!”

This pattern transition can also be seen in the raw-data time series of the items (comp. Figure 2: “resilience/ability to cope with stress” (**A**), “autonomy” (**B**), “withdrawal” (**C**), and “stress” (**D**), of which especially the last two show seemingly erratic fluctuations). The integration of all items into the two main standardized factors as shown in Figure 3, make the order-transition after almost half of the therapy much more evident. Also the mutual exclusive correlation pattern of the personality states (items of factors I and II, see Figure 5A) disappeared almost immediately after the order transition (Figure 5B). All this information was integrated into the ongoing therapy, clarifying the change in terms of state dynamics and related cognitions and emotions, both for the therapist, as well as for the client.

**Figure 6:**
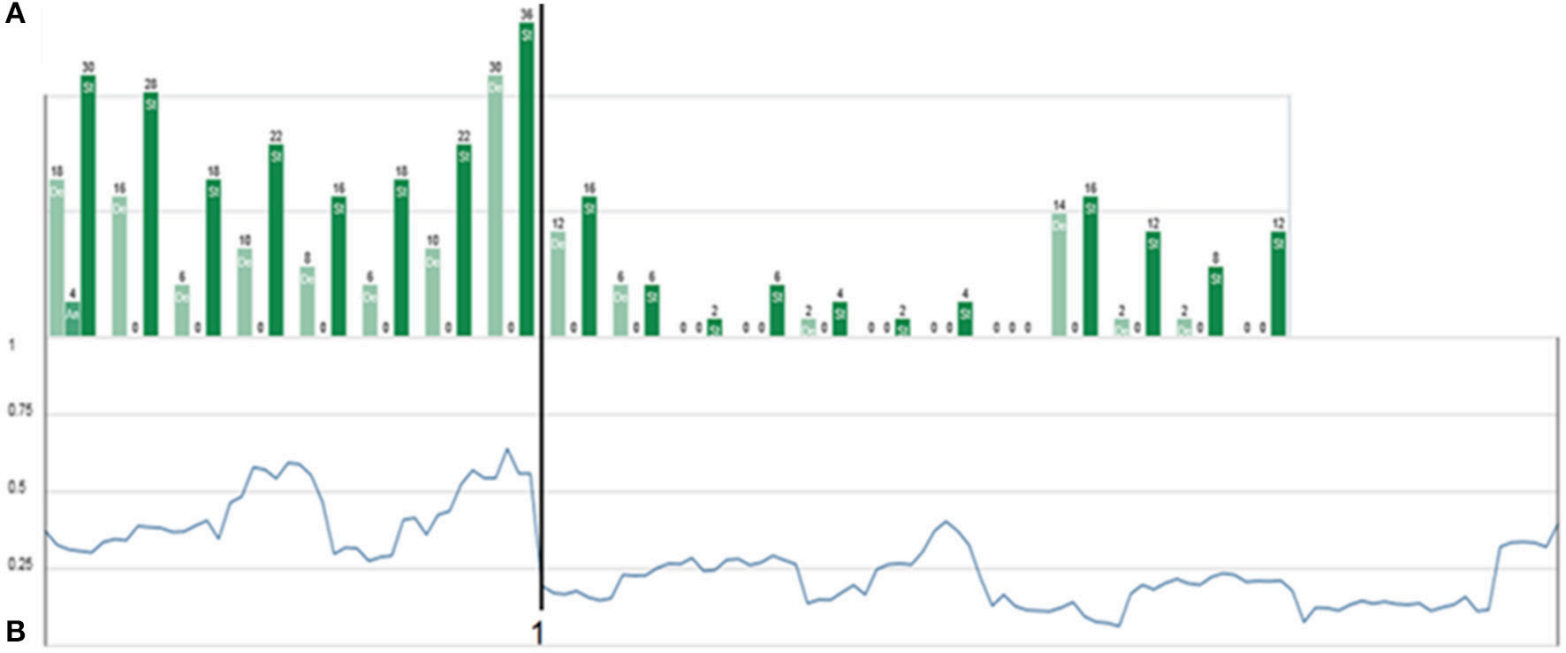
**Weekly DASS-21 progress and absolute inter-item correlation. (A)** Triplets of depression (light green), anxiety (medium green), and stress scores (full green), resulting from weekly assessment of the DASS-21 (Lovibond and Lovibond, 1995). Depression and stress are expressed strongly, while anxiety scores are surprisingly low. The order transition (flag 1) is characterized by an increase and sudden drop in stress and depression scores. **(B)** Mean absolute inter-item correlation of all possible correlations (averaging without respecting the sign of the correlation values) of the personalized 18 items of Mrs. A.'s individual questionnaire. The order transition is—in accordance with complex systems theory—preceded by a (local) maximum of the inter-item-correlation. The overall higher inter-item-correlation before flag 1 might be interpreted as the presence of an “enslaving” state dynamics, which reduces the degrees of freedom in the patient's life.

This crisis and the resolution thereof was accompanied by an increase of depression- and stress-scores (assessed by the weekly administered DASS-21, Lovibond and Lovibond, 1995), followed by a drop to low scores on these attributes just at the order transition (Figure 6A).

**Figure 7:**
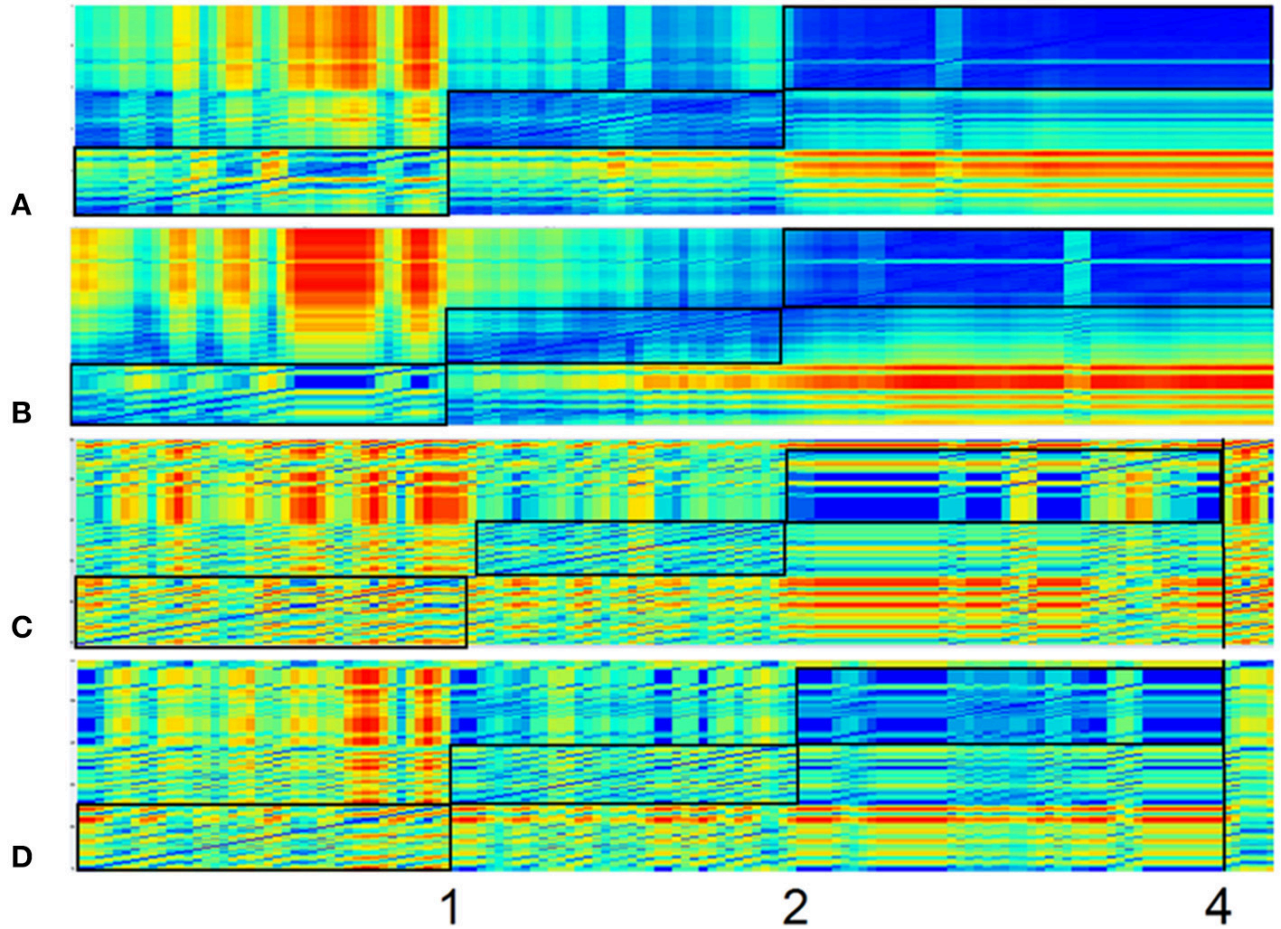
**Recurrence plots of four items**. Recurrence Plots of the items “resilience/ability to cope with stress” **(A)**, “autonomy” **(B)** (both factor II), “withdrawal” **(C)**, and “stress” **(D)** (both factor I). All plots are based on a 3-dimensional phase space embedding with time-delay τ = 1. Numbers 1 and 2 mark order transitions. Abscissa and ordinate both represent the time dimension, while the color of each pixel depicts the similarity (recurrence) of the item-values of respective time-snippets. This similarity/distance ε between two points of the trajectory in phase space is expressed as pixel with a continuous color scale, ranging from warm colors (yellow/red)—which denote discrepant values (transient)—to colder colors (blueish), which stand for similarity (recurrent) and have less distance between the vector points in the phase space. Framed blocs before marking 1, between 1 and 2, and after 2 can thus be understood as respective homogenous epochs.

A second change of pattern occurred some 5 weeks later (flag 2 in Figures 2–4, 7). Mrs. A. had bought presents for her partner's hobby, spending so much money that she had to ask her parents for financial support. She experienced that as regress to childhood, activating “child”-related states. It triggered a small crisis, which in contrast to the first crisis played out on a “high level.” She managed to utilize the crisis by means of the idiographic systems model to yet better understand her psycho-dynamics. In combination with the therapeutic sessions, she progressed from the crisis into a more stable pattern as can be visualized by many items of her questionnaire (see Figures 2–4, after flag 2).

Mrs. A. left the day-treatment clinic 10 days after resolving this second crisis. The dismissal as such appeared to not have caused any turbulence (Figures 2–4, flag 3). As bridging technology to aftercare, Mrs. A. chose to continue filling in the daily questionnaires. She reported that the routine of filling in helped her as self-referential support and in widening personal perspectives, creating a sense of stability. Just before the projected monitoring end, 7 weeks after dismissal and after 138 days in total, Mrs. A. experienced another crisis. An incidence in her partner's life reactivated own traumata and stressors (flag 4 in Figures 3C, 4A, 7). However, on her own accord, she managed to handle this crisis and be of help to her girlfriend.

Taken together, Mrs. A. reported that she was constantly motivated and profoundly benefitted from the work with an individualized questionnaire in combination with regular therapeutic sessions, which consequently and competently focused on patterns of her psychological functioning and the linkage of the involved variables as mapped with the idiographic system model. In contrast to an earlier hospital stay at the same clinic, where she filled in a generalized daily questionnaire only sporadically, she did not miss a single day of assessment in the personalized approach.

A complete picture of the therapy process including a 7-week long aftercare period is synoptically shown in Figures 4, 6, 7. Major order transitions are visualized in terms of the raw data resonance diagram and the complexity resonance diagram (Figures 4A,B). The period of strongest perturbation is to be found in the first part of the therapy, mainly expressed in items belonging to the “child”-related factor of stress and coping, as the mosaic-like warm colors of Figure 4B exemplify. This mirrors the erratic state dynamics of the period, on the one hand, and, on the other hand, the complexity and instability before her decision to let go her occupational goals (flag 1 in applicable figures). Approaching the solution of this crisis, Mrs. A. showed an intensification of stress- and depression-scores, as the weekly Depression-Anxiety-Stress-Scale (Figure 6A) shows. This pattern is often intuitively perceived by therapists as crisis before big decisions or seemingly “sudden gains” (e.g., Tschacher et al., 1998; Stiles et al., 2003; Hayes et al., 2007; Haken and Schiepek, 2010; Lutz et al., 2013; Schiepek et al., 2014).

The alternating dynamic of the two major states of Mrs. A. is also expressed in a high mean inter-item-correlation (the mean of all absolute correlations of the 18 items from the personalized questionnaire) in the first third of the monitoring period and before resolution of the first crisis (Figure 6B). The maximum inter-item-correlation appears just before the first order transition (flag 1), a phenomenon in line with observations in complex systems theory of increased synchronization of systems and their components during critical instabilities just before phase transitions occur (Haken, 2004; Scheffer et al., 2009; Haken and Schiepek, 2010, pp. 411ff.; Dakos et al., 2012). The factors or state dynamics might in this light be understood as an enslaving bi-stable attractor, from which Mrs. A.'s alternating behavior hardly escapes (see for a similar dynamics of “states of mind” in another case, analyzed by the method of configuration analysis (Horowitz, 1987; Beirle and Schiepek, 2002; Haken and Schiepek, 2010, pp. 328–343). After solving the first crisis, this pattern dissolves in favor of the desired “adult”-state (factor II) and accordingly a sharp drop in the pathological oversynchronization of the psychic system of Mrs. A. is visible in terms of a sudden decrease of the inter-item correlation (Figure 6B, flag 1; Figure 5B).

In Figure 7, the dynamics of the items “resilience/ability to cope with stress” (**A**), “autonomy” (**B**) (both factor II), “withdrawal” (**C**), and “stress” (**D**) (both factor I) (comp. Figure 2) are analyzed by the method of colored recurrence plots. Here the same three periods of recurrent dynamics and pattern transitions (discriminated by flags 1 and 2) can be identified.

## Discussion

The concept of synergetic process management, which entails a resource-focused interview, the development of an idiographic system model, an individual process questionnaire for daily online monitoring, regular therapeutic sessions with feedback based on the current data-profile, and ambulant aftercare with the Synergetic Navigation System as bridging technology, is a feasible approach also for patients with complex structural dissociation of personality. As such it enables clients' explicit learning processes, namely comprehending the systemic connections of psychological variables and their dynamics, thereby creating a competency for one's own system. Furthermore, implicit learning is promoted in terms of cooperation with the therapist, the act of making inner processes transparent and fostering own initiative, rendering psychotherapy not a giver-receiver relationship, but a participatory process management on eye level.

The approach of synergetic process management draws on a strong, meta-theoretical background of complex system theory. Hereby, one not only gains theoretical and practical tools, such as time series analysis techniques like recurrence plots or dynamic correlation matrices, but also opens the door for a combination of therapeutic applications. As a meta-theoretical concept it possibly overarches usage of systemic therapy (e.g., idiographic systems model), cognitive behavior therapy (e.g., *in vivo* desensitization interventions timed to critical transitions), trauma- and state-focussed approaches (e.g., Flatten, 2011; Nijenhuis, 2015, 2016), or any other psychotherapeutic tools. A better understanding of a client's psychological variables and processes will be beneficial to any form of psychotherapy, and just as well for patients with amnesias, state-specific attention patterns, or perceptual and cognitive deficits. These fields being impaired and simultaneously necessary for the solution —as is the nature of psychological problems—, makes it even more necessary to individualize the therapeutic approach, and consequently and repeatedly feedback information on processes (continuous cooperative process control). In that way clients are enabled to gain a meta-perspective of their own psychological patterns, increase understanding, and open the door for self-induced change. In cases such as the one presented, where dissociative states create an apparently erratic and confusing alternation of emotional and mental states (see Figures 2C,D), clients and their close one's stand dazzled when not supported. The tools of monitoring systems such as the SNS allow for capturing and analyzing ongoing processes, thereby boiling down seemingly irregular behavior to understandable patterns. Creating a client's idiographic system model provides consensual information, while the process-monitoring casts that information into a meta-perspective on dynamic patterns. From there, it is a feasible step to identify the patterns and systemic causes of states, making therapy thereof possible. For Mrs. A. it was, e.g., an astonishing fact that the open modeling work of her idiographic system led to two sets of items, which were rather clearly assignable to her two major personality states. A fact that helped her understanding herself, integrating differential aspects of her life into another and reversely validating her idiographic systems model. Understanding dissociative fragmentation of personality as the trauma-related correlates of fragmented neuronal networks (Flatten, 2011), it becomes evident that any work aimed at synthesizing these fragmented aspects will create understanding and a sense of coherence in affected clients. In order to do so, it seems advisable to allow the client to be the author of his or her own modeling work while entertaining a consensual form of dialog.

The therapeutic work with Mrs. A. utilized naturally occurring crises and transformed these into therapeutically relevant order transitions of emotional and mental processes. It is noteworthy to stress the point that these crises and the accompanying transitions were by no means induced by specific “interventions”. Here, therapy can be understood as a process that creates conditions for self-organized change (Schiepek et al., 2015).

One might argue that daily administration of questionnaires yields invalid results, which in fact might change the “object” of the research process (reactive measurement). We fully agree that creating an individualized questionnaire and its daily completion does change the respondent. It is however a change that is therapeutically relevant and intended, when clients improve in comprehending their own mental and behavioral processes. That ultimately will yield the most valid “scores” possible, because it inherits not only the change of psychological variables themselves, but also the change of their interconnectedness. In classical test-theory that might be understood as problematic (e.g., in terms of low test-retest reliability). However, it is therapeutically valid and useable when a client with dissociated states starts to merge these (e.g., as shown with the changing correlation matrices and the course of the averaged inter-item correlation).

Synergetic process management is a concept that sets the client and their needs into the focus of attention, asking for a cooperative, individualized, and meta-theoretical attitude of therapist and client. It then allows for the emergence of synergy effects, creates a basis for interdisciplinary case reviews, offers a bridging technology for changing therapeutic settings (e.g., from in-patient or day-treatment programs to out-patient after-care), and collects vast amounts of valid and reliable data, which will help the individual therapy at hand as well as psychological research in general.

## Author contributions

GS: Contributed to all aspects of the article, including concept, data gathering, creating tables and figures, writing and review of text; BS: Contributed to concept, data gathering and review of the text; WA: Contributed to concept and review of text. HS: Contributed to creating tables and figures and review of text; BA: Contributed to concept, creating tables and figures, writing and review of text.

### Conflict of interest statement

The authors declare that the research was conducted in the absence of any commercial or financial relationships that could be construed as a potential conflict of interest.
